# A systematic review on the modifications of extracellular vesicles: a revolutionized tool of nano-biotechnology

**DOI:** 10.1186/s12951-021-01219-2

**Published:** 2021-12-30

**Authors:** Alok Raghav, Goo-Bo Jeong

**Affiliations:** 1 Multidisciplinary Research Unit, Department of Health Research, MoHFW, GSVM Medical College, Kanpur, India 208002; 2 Department of Anatomy and Cell Biology, College of Medicine, Gachon University, 155 Getbeol-roYeonsu-gu, Incheon, 21999 Korea

**Keywords:** Synthesis, Extracellular vesicles, Nano-biotechnology, Tailoring, Drug delivery, Systematic review

## Abstract

**Background:**

Tailoring extracellular vesicles (EVs) can bequeath them with diverse functions and efficient performance in nano-biotechnology. Engineering and modification of EVs improves the targeted drug delivery efficiency. Here, we performed systematic review of various methods for EVs modifications.

**Methods:**

PubMed, Scopus, ISI Web of Science, EMBASE, and Google Scholar were searched for available articles on EVs modifications (up to March 2021). In total, 1208 articles were identified and assessed, and then only 36 articles were found eligible and included.

**Results:**

Six studies demonstrate the application of click chemistry, seven studies used co-incubation, two studies used chemical transfection, four studies implicated electroporation and sonication approach for modification of EVs. Moreover, two studies utilized microfluidics as suitable approach for loading cargo into EVs, while eight studies showed freeze–thaw method as feasible for these biological nanoparticles.

**Conclusion:**

Freeze–thaw approach is found to be convenient and popular among researchers for performing modifications in EVs for the purpose of targeted drug delivery loading. Clinical-grade EVs production with good clinical practices (GCPs) is challenging in the current scenario. More studies are needed to determine the best suitable approach for cargo loading of EVs that may be exploited for research and therapeutic use.

**Graphical Abstract:**

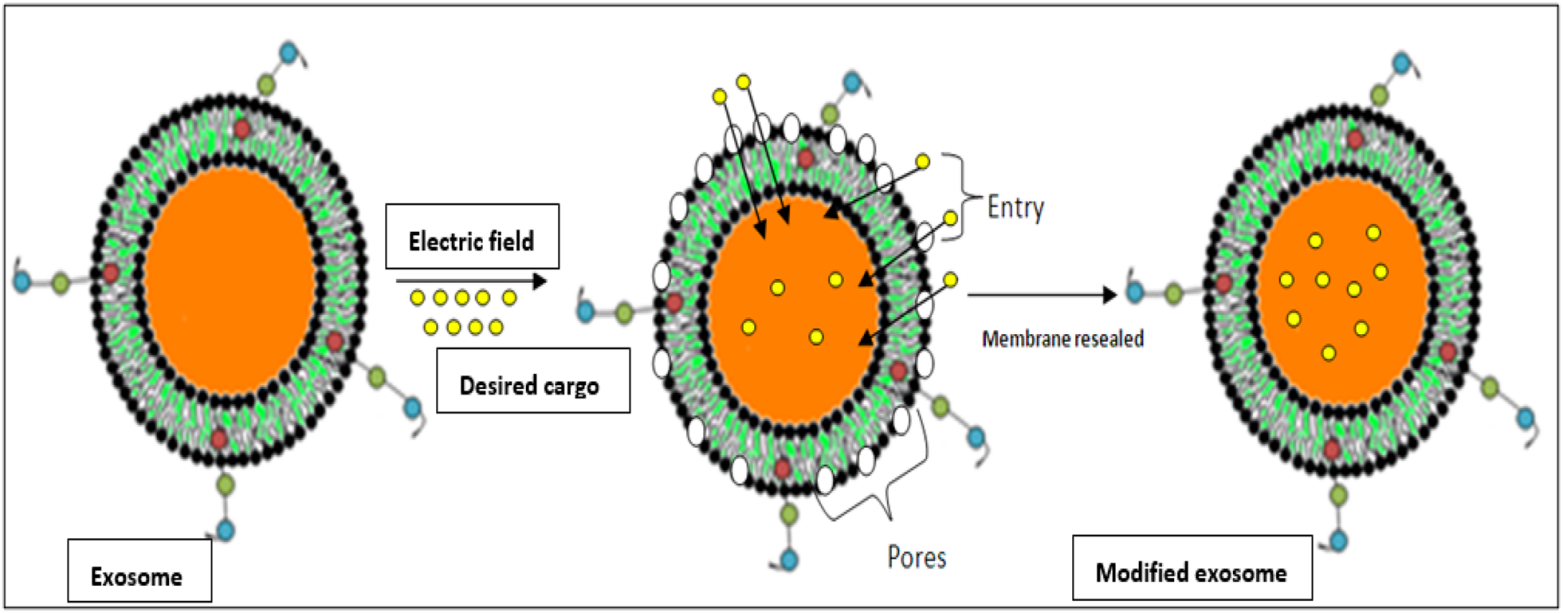

## Highlights

- Extracellular vesicles (EVs) can be modified using various methods available including physical, chemical and engineering based. These tailoring methods are helpful in targeting drug delivery to treat various diseases. Moreover, EVs have the ability to modify that’s due to presence of lipid bilayer membrane, that’s effectively participate in loading and unloading of desired drug.
- EVs expressed from the specific cell types can give useful information about the pathogenesis of a particular disease in the form of unique nucleic acids, protein and lipid sequences and therefore, EVs derived from these cells can be used as specific diagnostic biomarker for diagnosis of diseases.
- Modified EVs using various drugs or miRNAs can be used for targeted drug delivery to specific cells.

## Introduction

Extracellular vesicles (EVs) are naturally occurring nanoparticles of endosomal origin released from a wide variety of cells. They are membrane-bound vesicles ranging in size from 30–150 nm with a cup-shaped morphology, as seen under a scanning electron microscope (SEM), along with a floatation density of 1.10–1.21 g/mL in a sucrose gradient column [1]. The exosomal cargo is mostly rich in proteins (including tetraspanins (CD9, CD63, and CD 81), MHC class I and II, and heat shock proteins (HSP 60, 70, and 90), lipids, sugars, and nucleic acids [2]. The cargo imparts functionality to the EVs for different cellular communications, like paracrine, autocrine, endocrine and/or juxtacrine signaling [3], while surface proteins identify the EVs for cargo delivery.

Three types of EVs exist that are classified based on size and biogenesis, the smallest being exosomes (30–150 nm), followed by microvesicles and apoptotic bodies (larger than 100 nm). EVs are released into the extracellular environment after multivesicular bodies (MVBs) fuse with the plasma membrane, while microvesicles and apoptotic bodies are released directly by the plasma membrane of living and dying cells, respectively. EVs release involves various steps: the formation of early endosomes, the fusion of MVBs containing intraluminal vesicles (ILVs) with the plasma membrane by exocytosis, and the release of EVs in the extracellular space [4]. EVs are present in all bodily fluids secreted by cells, including blood [5], urine [6], plasma [7], breast milk [8], saliva [9], bile, synovial fluid, semen, amniotic fluid, ascites fluid (peritoneal cavity), and bronchoalveolar and gastrointestinal lavage fluid [10]. Biomolecules present both inside and on the surface of EVs are important markers for their isolation and characterization. As per the International Society for Extracellular Vesicles (ISEV), vesicles isolated from any type of extracellular fluids (i.e., any bodily fluids or cell culture-conditioned media) are referred to as EVs [11]. ISEV has also defined experimental guidelines for the characterization of EVs [11].

Liposomes, micelles, and polymer-based nanomaterials are the most commonly used drug delivery agents for clinical studies, and these have been curated over time to improve the solubility, efficacy, and stability of drugs [12]. However, clinical trials with these systems have been associated with limitations like immunogenicity, poor bio-distribution, and a short half-life. Therefore, to overcome these limitations, researchers have sought alternatives for delivery vehicles that are endogenous to the cells/tissue.

**Fig. 1:**
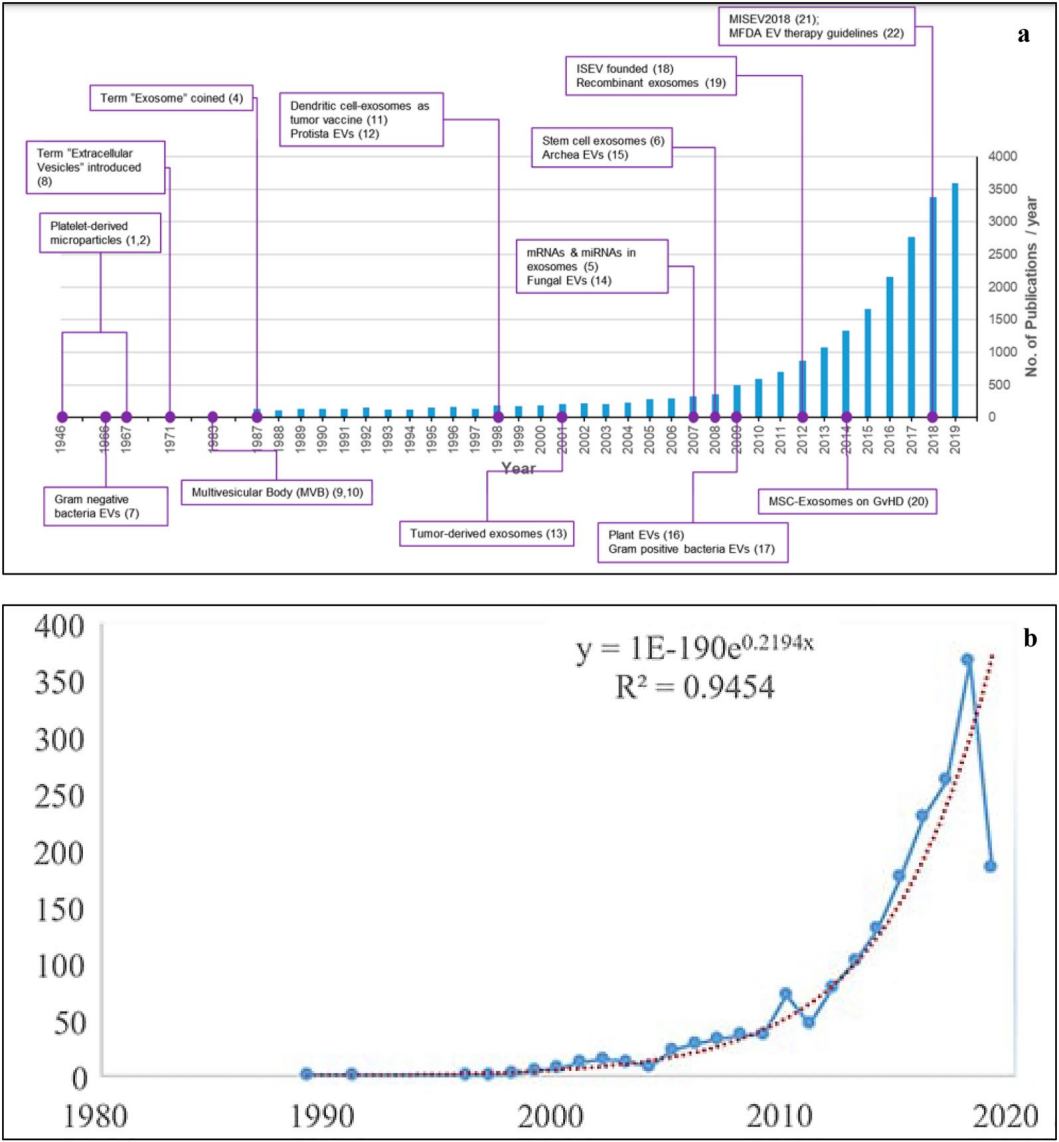
**a** Trends of publications and major discoveries regarding exosomes. **b** Publication trends in Exosome research field from 1989 to 2019 (red dotted line: the prediction trends, blue line: the original trends). (Adapted from Reference [62] and [63] respectively under open access article distributed under the Creative Commons Attribution License)

EVs have been exploited as drug delivery vehicles in several studies and possess an edge over the available drug delivery protocols in therapeutics [12–14]. Figure 1 depicts the increasing global trend in publications related to EVs research over the past 30 years (1989–2019). The dynamic composition of EVs allows for the tailoring of a “cargo of interest” for enhanced efficacy and specificity. Additionally, EVs may be modified for prolonged circulation time, specific target cell recognition using cell surface markers, negligible toxicity, and immune tolerance. EVs can be simultaneously manipulated with multiple types of deliverables, like drugs, proteins, and coding/non-coding nucleic acids. However, further studies are required to evaluate whether there exists any sort of allogeneic immune rejection among EVs from different donors and recipients [15, 16].

EVs have a diverse physiological role in the immune system, cell-to-cell interaction, central nervous system (CNS), tissue regeneration therapeutics, cardiovascular diseases (CVDs) and COVID-19 [17]. Moreover, the definite role of EVs remains elusive. In previously published literature it was found that EVs extracted from the dendritic cells (DCs) bearing peptide MHC-I, II, CD80/B7.1 and CD86/B7.2 induces immunomodulation through activation of CD4+ and CD8+ T-lymphocytes [17]. EVs were also found to mediate immunosuppressive effects on natural killer (NK) cells and T lymphocytes [17]. EVs derived from oligodendrocytes (ODCs) enriched with prime components of the myelin sheaths including myelin basic protein (MBP), myelin proteolipid protein (PLP) and oligodendrocyte glycoprotein (OG) along with several essential factors and protein that is needed for the vital functioning of axons [18]. In one of the previously published studies, ODCs derived EVs helps in protecting neurons from oxidative stress (OS) and control the biogenesis of the myelin sheaths (MS) [19]. EVs also play important role in angiogenesis and cardiovascular diseases (CVDs). In one of the previously published study, it was found that EVs derived from the CD34+ stromal cells showed a therapeutic effect on angiogenesis, both in-vitro and in-vivo [20]. In another human study, EVs showed a cardio-protecting role in myocardial ischemia (MI) reperfusion injury mediated through paracrine effects of these EVs [21]. This review predominantly focuses on recent approaches used for EVs modification and clinical applications of these EVs for therapy and diagnostics.

**Fig. 2:**
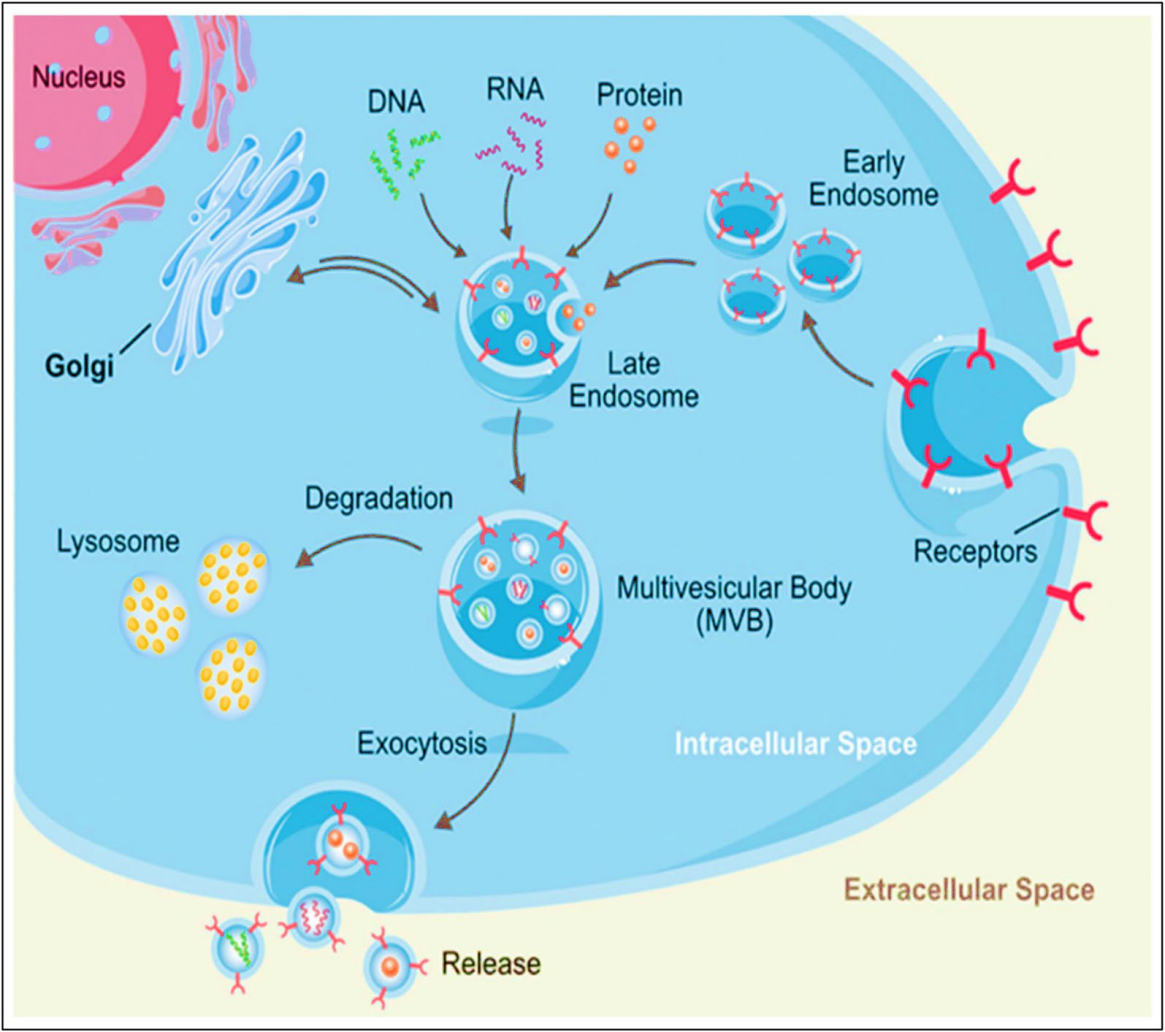
Pictorial representation of EVs biogenesis. MVB, multivesicular bodies. (Adapted from Ref No. [67] under Creative Commons Attribution License 4.0)

EVs biogenesis is a constitutive phenomenon that is initiated with an inward invagination of the plasma membrane, leading to the formation of early and late endosomes in the cytosol by the cells. The late endosome/MVB membrane invaginates further and forms ILVs inside large MVBs. Inward invagination of the endosomal membrane is accompanied by the engulfment of several proteins and numerous cytosolic components. Later, these MVBs fuse with the plasma membrane and release the EVs by exocytosis in the extracellular space, as depicted in Fig. 2. Previous studies have elucidated that MVB biogenesis [17], vesicle budding, and EVs cargo sorting are either dependent on or independent of the endosomal sorting complex required for transport (ESCRT), which is complex protein machinery consisting of four subunits, (0 to III). The ESCRT-mediated cargo sorting mechanism involves the identification and sequestration of ubiquitinated proteins to specific sites of the endosomal membranes. Further, sequential interaction with other subunits (I, II, and III) completes the complex and initiates the budding process. Sorting protein Vps4 helps in the detachment of the ESCRT III complex from the MVB membrane, leading to the formation of ILVs from cleaved buds. Meanwhile, the ESCRT-independent mechanism involves the interplay of proteins and lipids, such as tetraspanins (CD81), ceramides and others. Whether or not EVs biogenesis and cargo sorting will be ESCRT-dependent/independent primarily depends on the parent cell type responsible for EVs production, and this, in turn, is affected by the physiological status of the parent cells stimulated, stressed, or differentiated).

Diverse cell lineages secrete EVs with various cargo contents (proteins, lipids, and nucleic acids) [18]. A major proportion of the cargo comprises proteins, such as tetraspanins, heat shock proteins, cell adhesion proteins, membrane transport proteins, cell-signalling proteins, transcription proteins, and trafficking membrane fusion proteins. Moreover, lipid-profiling studies have revealed that phosphatidylserine (PS), phosphatidicacid, cholesterol, sphingomyelin (SM), arachidonic acid, prostaglandins, leukotrienes, and other fatty acids are some of the lipids commonly found in EVs. In addition to lipid components, EVs contain diverse non-coding RNAs, including miRNA, small nuclear RNA, small nucleolar RNA, long non-coding RNA, piwi-interacting RNA, rRNA, and tRNA [19–25].

In this systematic review, we present the currently available knowledge concerning suitable methods for the loading or delivering of the desired cargo into EVs that can be further translated for clinical purposes, such as in the treatment of various diseases. Our analysis provides evidence-based help for researchers and clinicians to exploit the field of EVs.

## Materials and methods

The present systematic review was framed following the Preferred Reporting Items for Systematic Reviews and Meta-Analyses (PRISMA) guidelines [26].

### Literature search

Published articles were selected from PubMed, Scopus, ISI Web of Science, EMBASE, and Google Scholar by searching for all available articles on modifications of EVs (up to March 2021). Searches were made using keywords: “extracellular vesicles” (Medical Subject Headings (MeSH) OR “Click Chemistry” (MeSH) OR “Co-Incubation” (MeSH) OR “Chemical transfection” (MeSH) OR “Electroporation” (MeSH) OR “Sonication” (MeSH) OR “Extrusion” (MeSH) OR “Freeze–thaw” (MeSH) OR “Genetic engineering” (MeSH) OR “Microfluidics” (MeSH). Two investigators screened the titles, aims and abstracts of the published articles to determine eligible articles for the present study. The same investigators evaluated full-length articles, and inclusion and exclusion criteria were applied to each article. Moreover, the same investigators screened the references of the initial eligible articles to identify all eligible articles for inclusion in the final list.

### Inclusion and exclusion criteria

Research papers screened during the literature search followed the following inclusion criteria for the present study: (1) Modification of EVs; (2) laboratory-based modification of EVs; (3) loading of drugs into the EVs, and (4) published an original article with all full-text literature and properly cited references. The following studies were excluded: (1) insufficient reported data with non-cited references; (2) published conference proceedings; (3) published review articles, letters, or text not in the English language; or (4) repetition of previously published articles.

### Data extraction

The author’s names, year of publication, country of origin, and types of methods used to modify the EVs were extracted. Outcomes were noted in the form of a best-suited method for the modification of EVs that may have translational potential in clinical setup under GCP guidelines [27].

## Results

**Fig. 3:**
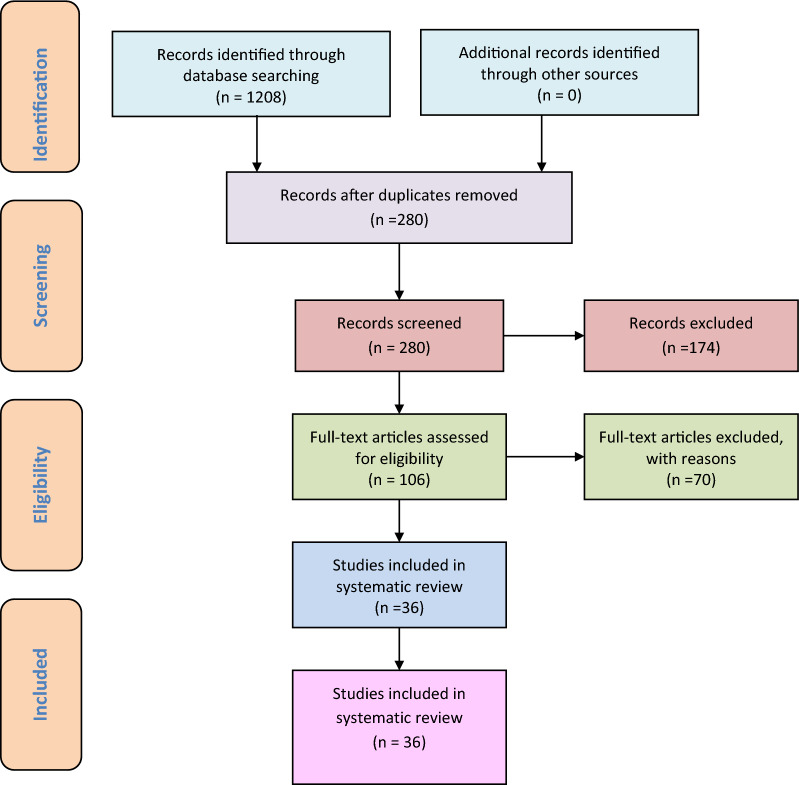
PRISMA flowchart

**Fig. 4:**
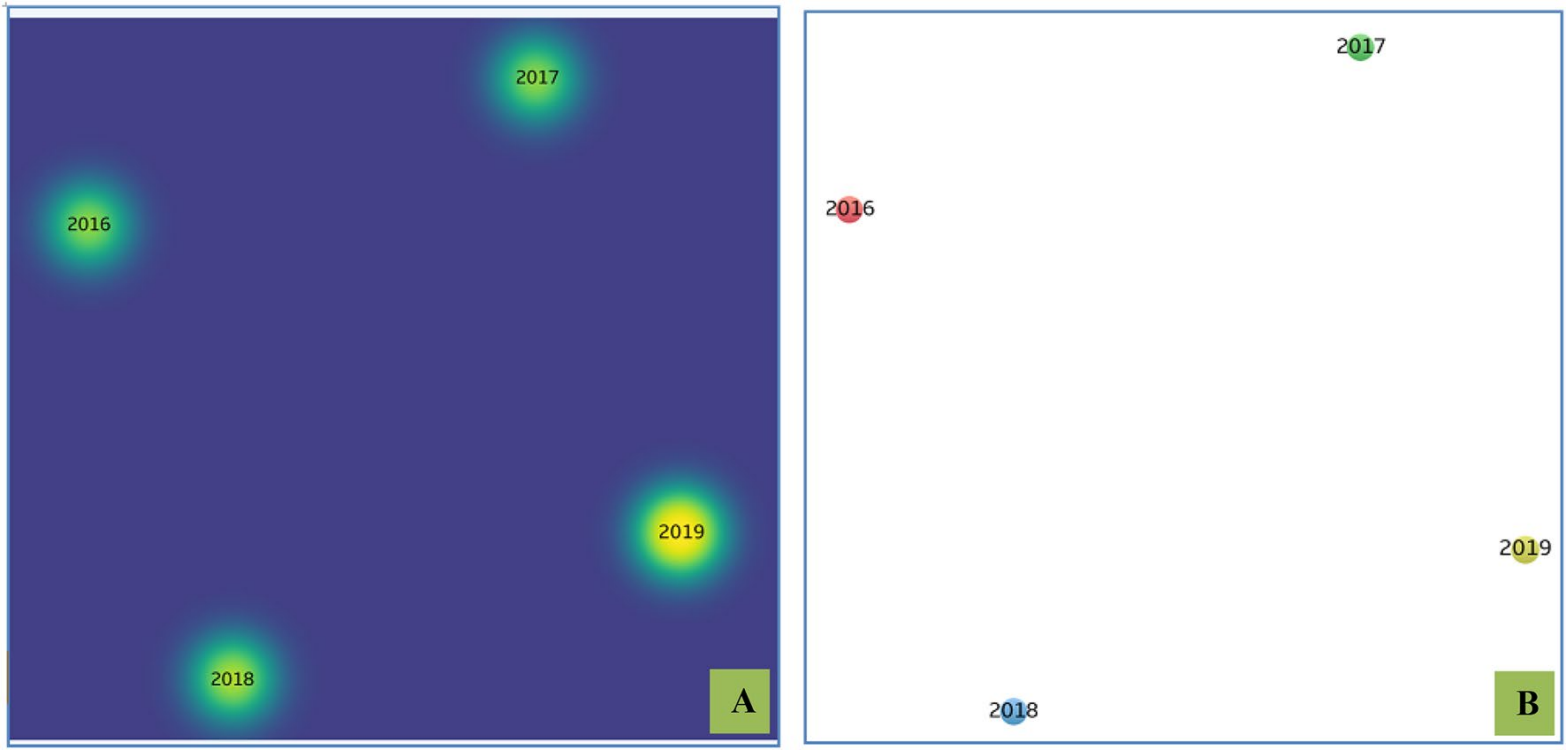
**A** Network visualization of the studies distributed in the systematic review associated with the exosomal modifications. **B** Density visualization of the studies distributed in the systematic review

A total of 1208 articles were screened; among them, 280 non-duplicate publications were identified. After the screening of titles and abstracts, 174 publications were excluded; then, another 70 published papers were excluded after the full-text screening of 106 published articles. The remaining 36 studies were included in this systematic review (Fig. 3). Network and density visualization of the studies included in this systematic review is demonstrated in Fig. 4.

### Study characteristics

Study characteristics of the eligible studies showed that most authors performed EVs modification and functionalization using the freeze–thaw method and co-incubation approach (Table 1). Seven studies used co-incubation to produce functional EVs that have a pioneering role in the treatment of various diseases, like cancer and other immunological complications. Moreover, eight eligible studies exploited the freeze–thaw approach for the surface modification of EVs. Click chemistry (CC) [27–32], freeze-thawing, co-incubation [15, 33–38]. Two studies used chemical transfection-based modification, which thus demonstrated the least preferred method for EVs modifications [39–44]. There were equal numbers of electroporation-assisted [41–44] and sonication approach-based [45–54] studies contributing four eligible studies each for EVs modification. Genetic engineering (GE) is gaining popularity as they are a widely used approach for EVs modification [55–59]. Moreover, microfluidics, a recently developed approach for EVs modification, showed the least number of studies (only two within the last 10 years) [60, 61].

**Table 1:** Summary of characteristics of included studies

No.	Title of the study	Authors	Place	Year	Method	References
1	Surface functionalization of exosomes using click chemistry	Tyson S, Krastina P, Nicole MP, Indushekhar P, Jasmina S. Redzic, M. Graner, Peter SJ, Thomas JA	United States	2014	Click Chemistry	[27]
2	In situ one-step fluorescence labeling strategy of exosomes via bioorthogonal click chemistry for real-time exosome tracking in vitro and in vivo	Sukyung S, Man KS, Seungho L, Yujeong M, Suah Y, Jinseong K, Yeonsun H, Hong YY, In-San K, Kwang YH and Kwangmeyung K	Republic of Korea	2020	Click Chemistry	[28]
3	Surface functionalized exosomes as targeted drug delivery vehicles for cerebral ischemia therapy	Tian T, Zhang HX, He CP, Fan S, Zhu YL, Qi C, Huang NP, Xiao ZD, Lu ZH, Tannous BA, Gao J	China	2018	Click Chemistry	[29]
4	Facile metabolic glycan labeling strategy for exosome tracking	Lee TS, Kim Y, Zhang W, Song IH, Tung CH	Republic of Korea	2018	Click Chemistry	[30]
5	Systematic quantification of the dynamics of newly synthesized proteins unveiling their degradation pathways in human cells	Ming T, Johanna MS, Haopeng X, Ronghu W	United States	2014	Click Chemistry	[31]
6	Integrating protein engineering and bioorthogonal click conjugation for extracellular vesicle modulation and intracellular delivery	Wang M, Altinoglu S, Takeda YS, Xu Q	United States	2015	Click Chemistry	[32]
7	Liposome co-incubation with cancer cells secreted exosomes (extracellular vesicles) with different proteins expressions and different uptake pathways	Emam, SE., Ando, H., Lila, ASA. Sherif EE, Hidenori A, Lila ASA, Taro S, Keiichiro O, Yu I, Mahmoud AM, Fakhr-eldin SG, Ikuko S, Tatsuhiro I	Japan	2018	Co-incubation	[33]
8	Focused ultrasound-augmented targeting delivery of nano-sonosensitizers from homogenous exosomes for enhanced sonodynamic cancer therapy	Xiaobing W, Yichen L, Lianmei B, Kaili G, Yali J, Kun Z, Quanhong L, Pan W	China	2019	Co-incubation	[34]
9	In vitro cultured human endometrial cells release extracellular vesicles that can be uptaken by spermatozoa	Valentina M, Elisa G, Sofia M, Natasa Z, Massimo C, Andrea S, Riccardo V,Paola V	Italy	2020	Co-incubation	[35]
10	Treatment of brain inflammatory diseases by delivering exosome encapsulated anti-inflammatory drugs from the nasal region to the brain	Zhuang X, Xiang X, Grizzle W, Sun D, Zhang S, Axtell R. C, Ju S, Mu J, Zhang L, Steinman L, Miller D, Zhang HG	USA	2011	Co-incubation	[15]
11	Exosomes derived from oviduct cells mediate the EGFR/MAPK signaling pathway in cumulus cells	Lee SH, Oh HJ, Kim MJ, Lee BC	Republic of Korea	2019	Co-incubation	[36]
12	Functional delivery of lipid-conjugated siRNA by extracellular vesicles	Aisling JOL, Imre M, Olivier G. de J, Miguel AV, Raymond MS, Samir EA, Matthew JAW, Pieter V	United Kingdom	2017	Co-incubation	[37]
13	Immune modulatory function of abundant immune-related microRNAs in microvesicles from bovine colostrum	Qi S, Xi C, Jianxiong Y, Liang L, ChenYZ, Ke Z	China	2013	Co-incubation	[38]
14	Delivery of small interfering RNA to inhibit vascular endothelial growth factor in zebrafish using natural brain endothelia cell-secreted exosome nanovesicles for the treatment of brain cancer	Tianzhi Y, Brittany F, Bret L, Salma A, Thuy P, Leanne L,Shuhua B	USA	2017	Chemical Transfection	[39]
15	Exosome–liposome hybrid nanoparticles deliver CRISPR/Cas9 system in MSCs	Yao L, Jiahua W, Weihuai GY, HuangZT, Lijia H, Jiali T	China	2018	Chemical Transfection	[40]
16	Cancer-derived exosomes as a delivery platform of CRISPR/ Cas9 confer cancer cell tropism-dependent targeting	Kim SM, Yang Y, Oh SJ, Hong Y, Seo M, Jang M	Republic of Korea	2017	Electroporation	[41]
17	Development of exosome-encapsulated paclitaxel to overcome MDR in cancer cells	Kim MS, Haney MJ, Zhao Y, Mahajan V, Deygen I, Klyachko NL, Inskoe E, Piroyan A, Sokolsky M, Okolie O, Hingtgen SD, Kabanov AV, Batrakova EV	USA	2016	Electroporation	[42]
18	Improved loading of plasma-derived extracellular vesicles to encapsulate antitumor miRNAs	Pomatto MAC, Bussolati B, D'Antico S, Ghiotto S, Tetta C, Brizzi MF, Camussi G	Italy	2019	Electroporation	[43]
19	Active loading into extracellular vesicles significantly improves the cellular uptake and photodynamic effect of porphyrins	Fuhrmann G, Serio A, Mazo M, Nair R, Stevens MM	UK	2015	Electroporation	[44]
20	Engineering hybrid exosomes by membrane fusion with liposomes	Sato YT, Umezaki K, Sawada S, Mukai SA, Sasaki Y, Harada N, Shiku H, Akiyoshi K	Japan	2016	Freeze–Thaw	[45]
21	Engineering exosomes as refined biological nanoplatforms for drug delivery	Luan X, Sansanaphongpricha K, Myers I, Chen H, Yuan H, Sun D	USA	2017	Freeze–Thaw	[46]
22	Potential therapeutic effects of exosomes packed with a miR-21-sponge construct in a rat model of glioblastoma	Monfared H, Jahangard Y, Nikkhah M, Mirnajafi-Zadeh J, Mowla SJ	Iran	2019	Freeze–Thaw	[47]
23	Donor dendritic cell-derived exosomes promote allograft-targeting immune response	Liu Q, Rojas-Canales DM, Divito SJ, Shufesky WJ, Stolz DB, Erdos G, Sullivan ML, Gibson GA, Watkins SC, Larregina AT, Morelli AE	USA	2016	Freeze–Thaw	[48]
24	Post-production modifications of murine mesenchymal stem cell (mMSC) derived extracellular vesicles(EVs) and impact on their cellular interaction	Le SS, Aarrass H, Lai KHJ, Bron P, Armengaud J, Miotello G, Bertrand-Michel J, Dubois E, George S, Faklaris O, Devoisselle JM, Legrand P, Chopineau J, Morille M	France	2020	Freeze–Thaw	[49]
25	The Immune Activity of PT-Peptide Derived from Anti-Lipopolysaccharide Factor of the Swimming Crab Portunustrituberculatus Is Enhanced when Encapsulated in Milk-Derived Extracellular Vesicles	Lee BH, Chen BR, Huang CT, Lin CH	Taiwan	2019	Freeze–Thaw	[50]
26	Exosomes as drug delivery vehicles for Parkinson’s disease therapy	Matthew JH, Natalia LK, Yuling Z, Richa G, Evgeniya GP, Zhijian H, Tejash P, Aleksandr P, Marina S, Alexander VK, Elena VB	Russia	2015	Freeze–Thaw, Sonication	[51]
27	Cytochalasin-B-inducible nanovesicle mimics of natural extracellular vesicles that are capable of nucleic acid tranfer	Oshchepkova A, Neumestova A, Matveeva V, Artemyeva L, Morozova K, Kiseleva E, Zenkova M, Vlassov V	Russia	2019	Freeze–Thaw, Sonication	[52]
28	Engineering macrophage-derived exosomes for targeted paclitaxel delivery to pulmonary metastases: in vitro and in vivo evaluations	Kim MS, Haney MJ, Zhao Y, Yuan D, Deygen I, Klyachko NL, Kabanov AV, Batrakova EV	USA	2018	Sonication	[53]
29	Paclitaxel incorporated exosomes derived from glioblastoma cells: comparative study of two loading techniques	Salarpour S, Forootanfar H, Pournamdari, M. Meysam AZ, Marzie E, Abbas P	Iran	2019	Sonication	[54]
30	Exosome-based tumor antigens adjuvant co-delivery utilizing genetically engineered tumor cell-derived exosomes with immunostimulatoryCpG DNA	Morishita M, Takahashi Y, Matsumoto A, Nishikawa M, Takakura Y	Japan	2016	Genetic Engineering	[55]
31	Exosomes derived from bone marrow mesenchymal stem cells overexpressing microRNA-25 protect spinal cords against transient ischemia	Zhao L, Jiang X, Shi J, Gao S, Zhu Y, Gu T, Shi E	China	2019	Genetic Engineering	[56]
32	Engineered exosomes for targeted transfer of siRNA to HER2 positive breast cancer cells	Limoni SK, Moghadam MF, Moazzeni SM, Gomari H, Salimi F	Iran	2019	Genetic Engineering	[57]
33	Delivery of siRNA to the mouse brain by systemic injection of targeted exosomes	Alvarez-EL, Seow Y, Yin H, Betts C, Lakhal S, Wood M	UK	2011	Genetic Engineering	[58]
34	Exosomes engineered to express a cardiomyocyte binding peptide demonstrate improved cardiac retention in vivo	Mentkowski KI, Lang, JK	USA	2019	Genetic Engineering	[59]
35	Microfluidic fabrication of cell-derived nanovesicles as endogenous RNA carriers	Wonju J, Dayeong J, Junho K, Siwoo C, Su C. J, Chungmin H, Ji YK, Yong SG, Jaesung P	Republic of Korea	2014	Microfluidics	[60]
36	Microfluidic on-demand engineering of exosomes towards cancer immunotherapy	Zhao Z, McGill J, Gamero KPP, He M	USA	2019	Microfluidics	[61]

### Systematic review

**Fig. 5:**
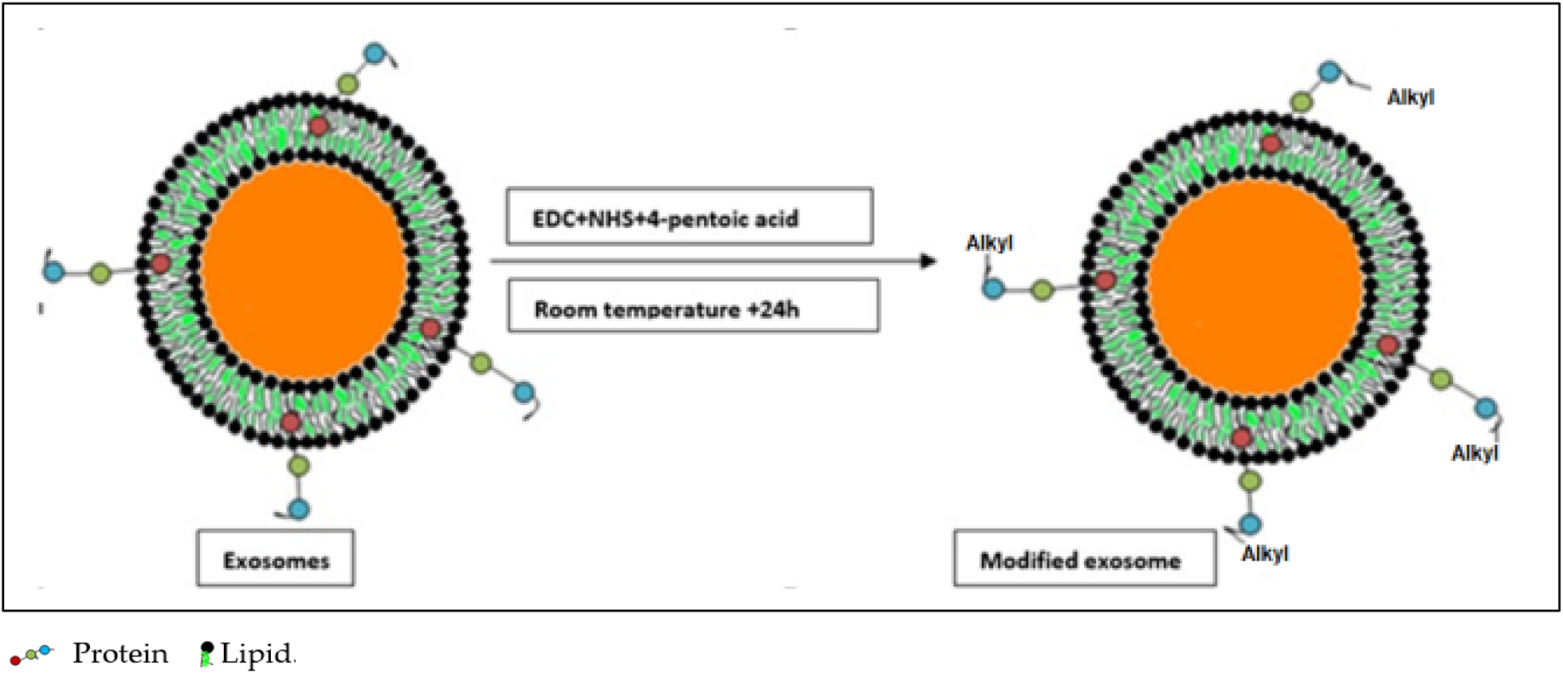
Surface modification of EVs using click chemistry reactions depicting the addition of alkyl groups to the EVs surface. EDC: N-(3-(Dimethylamino)propyl)-N-ethylcarbodiimide, NHS: N-hydroxysuccinimide

Six studies exploited the CC approach for the modification of EVs [15, 31–35]. Two studies published in 2014 followed by one published in 2015 used a CC approach for the surface functionalization of EVs for targeted drug delivery. In one of the published studies, authors used copper-catalyzed azide-alkyne cyclo-addition of azide-fluor 545 with EVs to assess internalization [27]. The authors further noticed that a 50 kDa exosomal protein efficiently binds 1.5 alkyne groups on its surface [27]. In a similar recently published study, the authors labeled the EVs surface with fluorescent probes using a CC approach to monitor the real-time tracking of EVs uptake within cells [28]. Similarly, CC has been exploited by authors for drug targeting, labeling of the EVs surface, and attachment of probes for tracking [27–32]. The CC approach is quite popular and still is in use currently. CC is depicted in Fig. 5.

Click chemistry is associated with several advantages and disadvantages. The advantages being, it is cost-effective and can be initiated in water without the need for a deoxygenated environment. The absence of non-aromatic double bonds within bio-macromolecule restricts undesirable side chain reaction occurrence. This method is environmentally friendly. The main limitation associated with the click chemistry is the reducing agent that might reduce Cu+ 2 to Cu0. This may be overcome by the incorporation of optimum concentration of reducing agent to catalyst ratio or using copper-stabilizing agents such as native cyclodextrins and tris- (hydroxyl propyltriazolylmethyl) amine (THPTA). Adverse impact on the human body is also among the main biological limitation of this approach. Excessive use of copper may lead to several associated side effects including neurological disorders. The main cause of toxicity associated with copper is the chemistry behind the reaction. Copper can readily accept and donate single electrons to change oxidation states thereby initiating in-vivo reduction of hydrogen peroxide to hydroxyl free radicals.

**Fig. 6:**
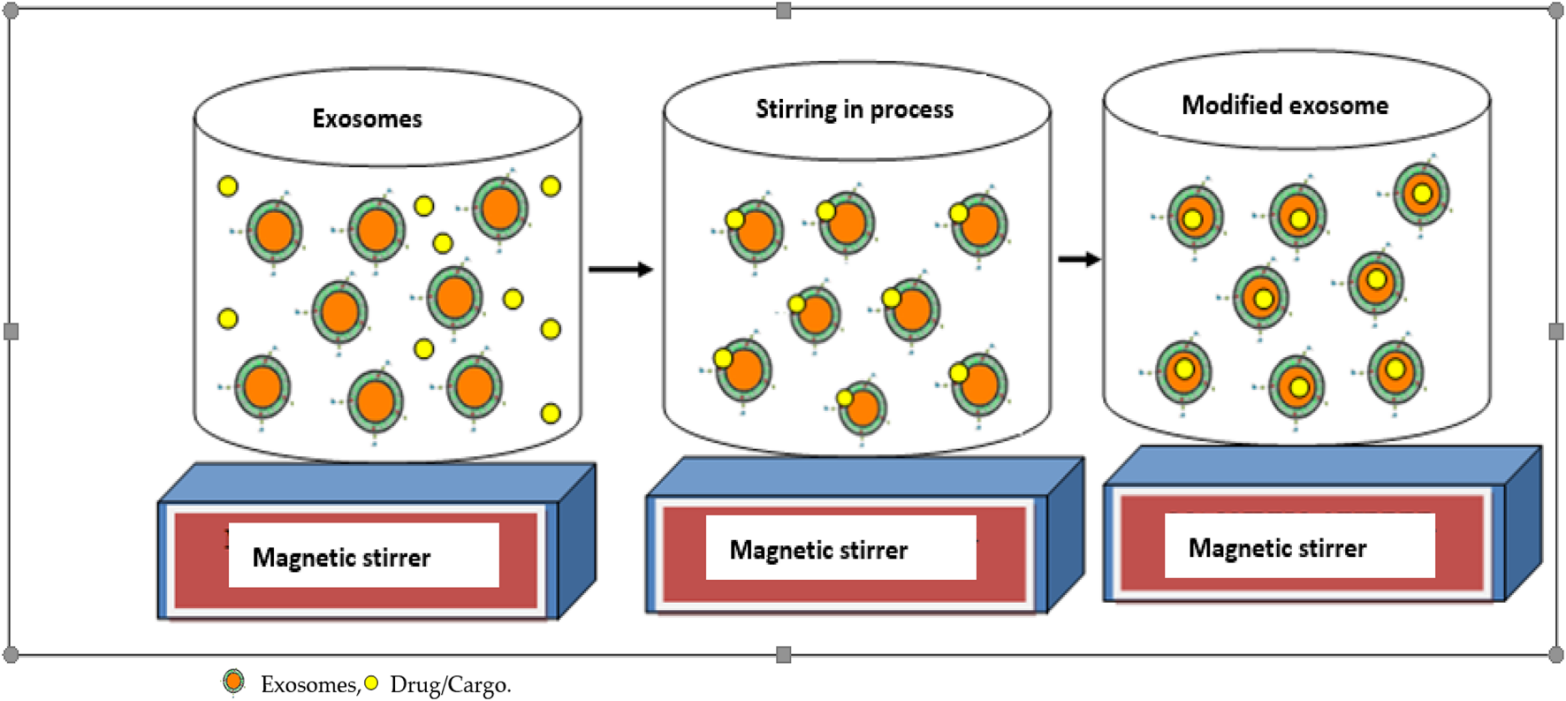
Modification of EVs using co-incubation of EVs with the drug or cargo upon continuous stirring

Co-incubation for exosomal modification is a popular method for dealing with nano-vesicles. In the present systematic review, seven studies used the co-incubation approach for the modification of EVs [15, 33–38]. In the co-incubation method, no external factor is applied to mediate the modification process. Figure 6 depicts the co-incubation method used for loading the target drug into EVs using a simple stirring process. In one study, the authors loaded sinoporphyrin sodium into EVs using the co-incubation approach, which was later used in therapeutic and imaging applications [34]. In another study on co-incubation, motile fluorescent sperm was noticed upon 48 h co-incubation with Vybrant dye-labeled EVs derived from endometrial cells [35]. In another study, EVs were loaded with curcumin using the co-incubation method for the treatment of brain inflammatory disease [15]. In another study, an engineering approach has been described in the modification of EVs [36]. Though proven to be useful for modification of EVs for their enhanced functionality with the desired cargo, this method is associated with some limitations as it affects the size of EVs, results in low yield, low entrapment and uncontrollable drug loading. It is a time-consuming approach due to passive diffusion. In this approach, a large amount of drug or cargo is needed to be delivered into the EVs. Hydrophilic cargo also faces problems in loading due to the outer lipid membrane of the EVs. However, it is a safe approach as it is non-toxic.

**Fig. 7:**
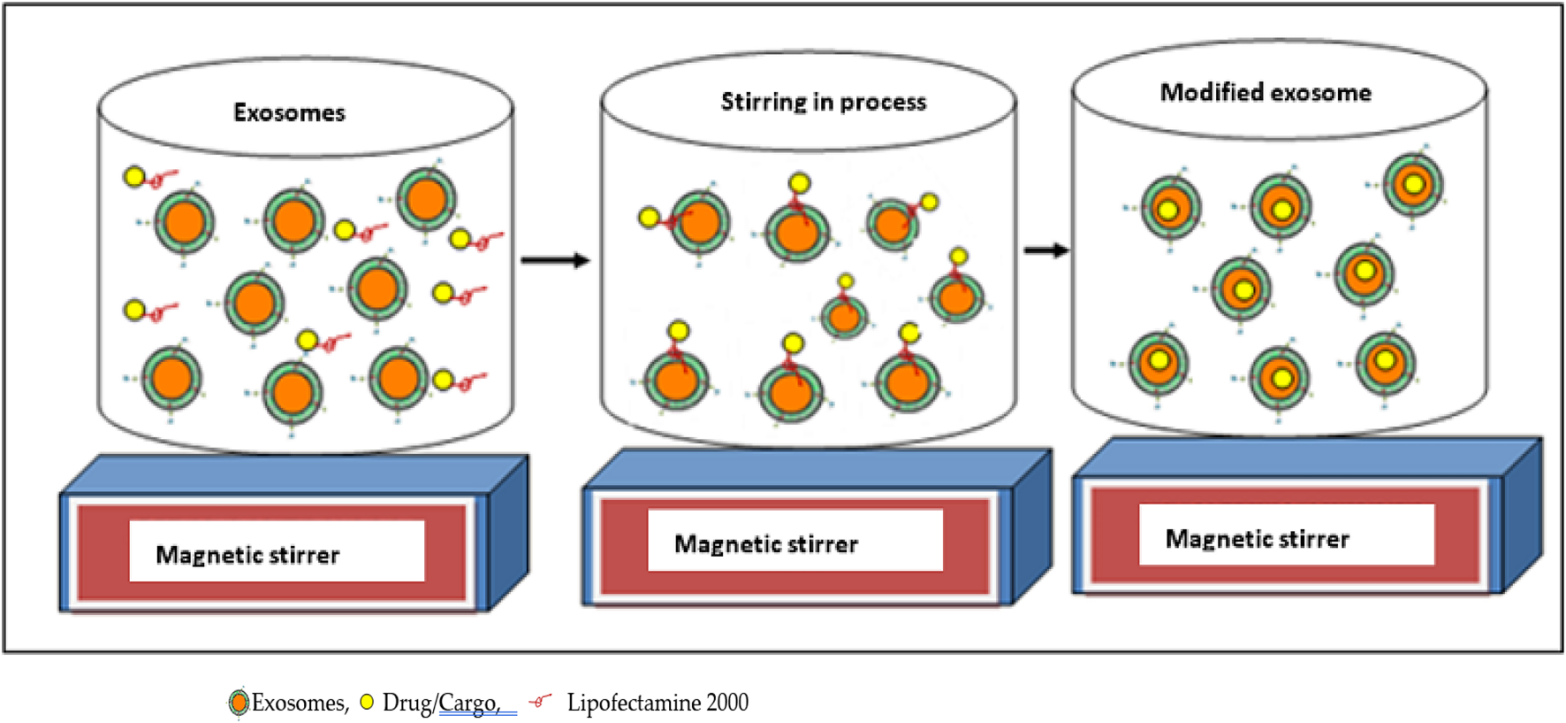
Modification of EVs using a chemical transfection agent, such as lipofectamine 2000

Chemical transfection or lipofectamine transfection-based modification offers limited choices for using this safer approach. Only two studies claimed to exploit chemical transfection for exosomal modifications [39, 40]. In these two studies, authors used chemical transfection to deliver siRNA and the CRISPR/Cas9 system, respectively [39, 40]. This approach is depicted in Fig. 7. Another advantage of using transfection agents like lipofectamine 2000 is that it can transfect a wide range of different cell types for a stable and longer duration. Moreover, the cons of this approach are cellular toxicity leading to high cell mortality along with immune rejections. Also, lipofectamine 2000 shows autofluorescence which might be interfering in fluorescence studies of delivering cargos. This approach is quite expensive and can’t be recommended for scale-up implications.

**Fig. 8:**
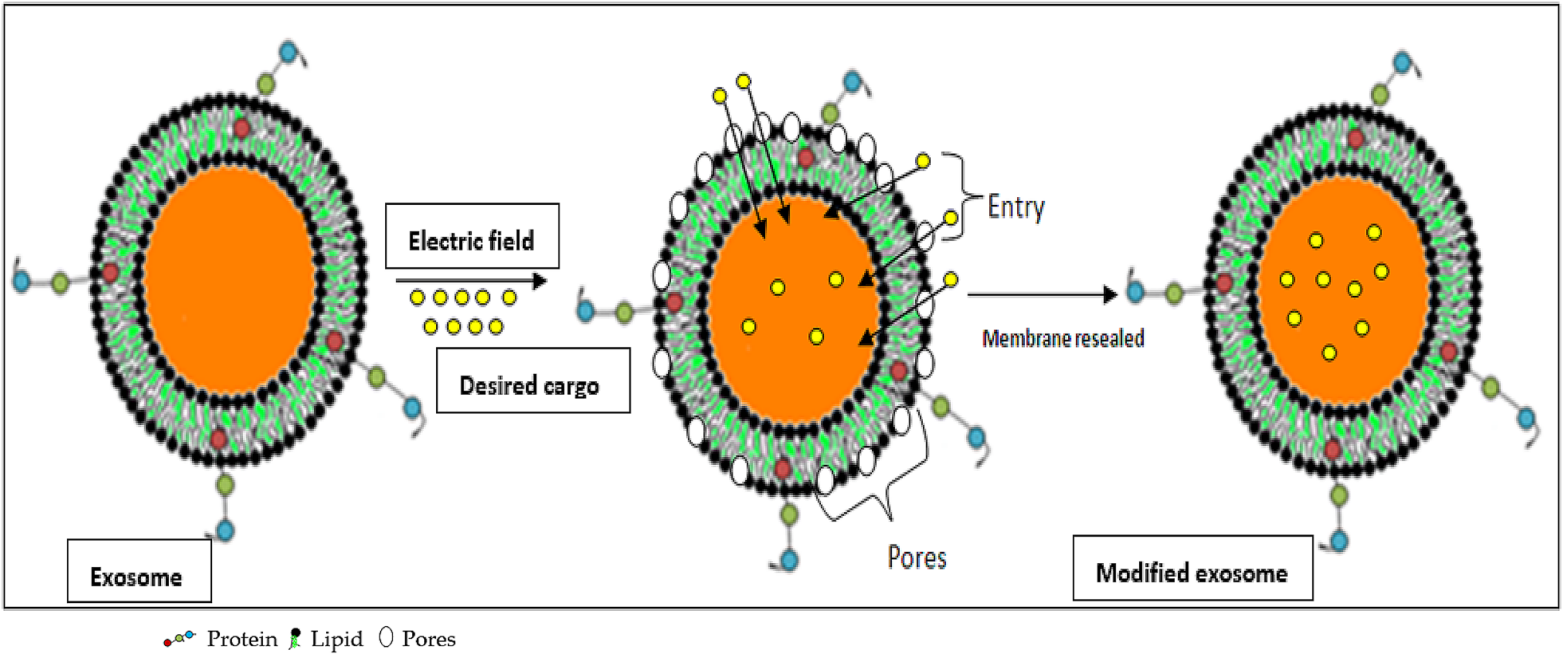
Depiction of electroporation technique for delivering the desired cargo inside EVs through pores created under application of electric field

Electroporation is another preferred method for the modification or loading of EVs for therapeutic and diagnostic purposes. In this method, an electrical field is applied to initiate the formation of pores in the lipid envelope of EVs in which the cargo of interest is placed; then, the membrane is resealed. Figure 8 shows a representation of the electroporation approach for loading of desired cargo. Four studies exploited the electroporation method [41–44]. In one of these studies, CRISPR/Cas9 was loaded into EVs using electroporation [41], and another loaded the drug paclitaxel into EVs for cancer treatment [42]. Similarly, other studies mentioned in this systematic review used electroporation to modify EVs for the treatment of various cancers [43–45]. Electroporation is a convenient approach for large-sized molecules like siRNA that may not passively be loaded into the EVs using co-incubation of chemical transfection. Smaller sized cargo can be efficiently incorporated through this approach. However, some limitations are also associated with this approach such as aggregation of siRNA and EVs due to the application of electric current thereby reducing the loading efficiency of the system. Moreover, an electric current of higher intensity may cause non-reversible disturbance in the phospholipid bilayer thus disrupting the structural integrity of the EVs leading to instability of these vesicles. It is necessary to optimize the electric current application according to the size, nature and surface charge of the cargo that has to be delivered inside the EVs.

**Fig. 9:**
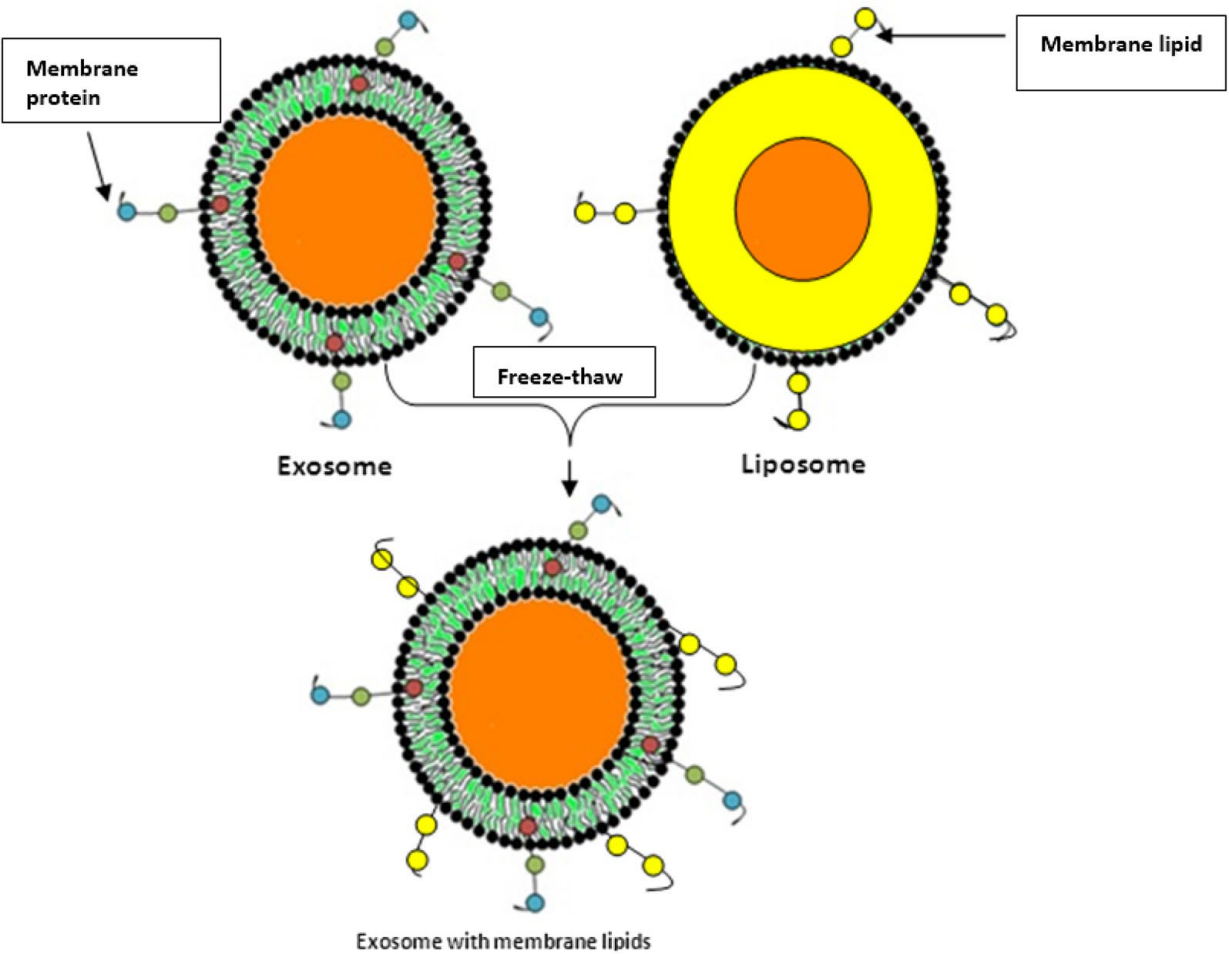
Freeze–thaw approach of EVs surface modification

Eight studies used a freeze–thaw method for EVs modification [46–53]. Using freeze-thawing, Sato et al. fused EVs with liposomes to make engineered hybrid EVs [46]. Figure 9 depicts the fusion of liposomes and EVs through the freeze–thaw approach. Other studies also used this modification approach for a variety of applications, including cancer treatment, labeling, and loading of the desired cargo or drug into EVs [47–53]. This is the most widely used, preferred, and accepted method of EVs modification applied by researchers for drug targeting and therapeutics. The main advantage of this method is the maintenance of exosomal membrane integrity throughout the cargo loading process. Animal cells derived EVs shown stability at low temperatures and also the repeated freeze–thaw cycles cannot affect the physicochemical and structural properties. However, sometimes more cycles of repeated freeze–thaw may lead to aggregation of EVs thereby contributing to the broad size distribution of cargo. This approach has low efficiency of cargo loading compared to the sonication and extrusion approach. Repeated freeze–thaw cycles may also hamper the fluorescence related study for the EVs due to the lipid dilution ratio.

Sonication is also among the preferable methods for loading drugs into EVs and surface modification. Four studies utilized sonication for the modification of EVs for various purposes [52–55]. The sonication method of loading cargo into EVs is suitable for biological molecules like small RNAs. This method has high loading efficiency too. Certain limitations like EVs membrane deformation, development of shearing forces, heat generation during the sonication cycle, loosing of EVs surface proteins and non-suitability for hydrophobic drug delivery are associated with this approach.

**Fig. 10:**
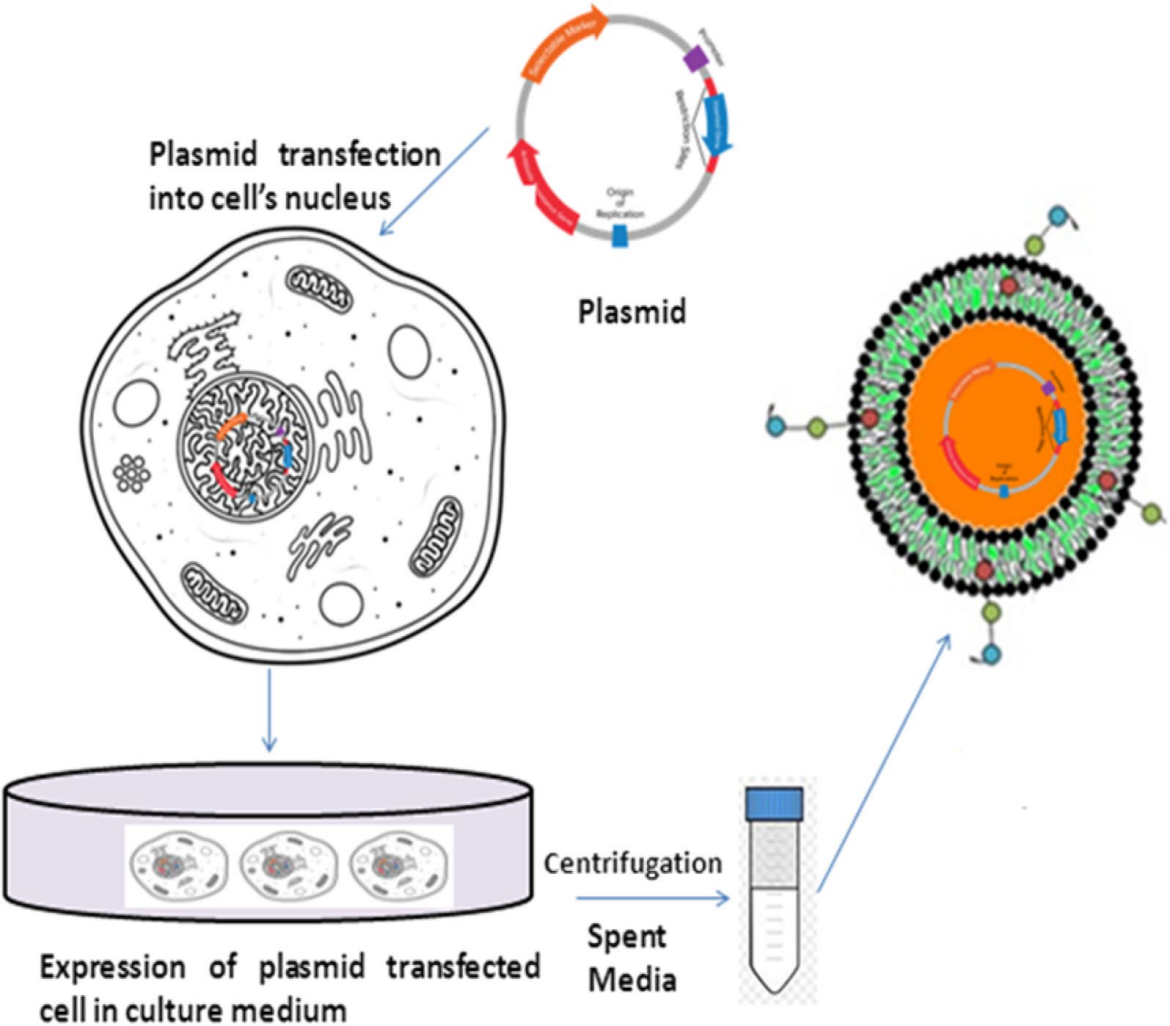
Strategy for engineering EVs displaying targeting ligands. The figure is showing engineering approach for modification of EVs. The plasmid having desired gene of interest is inserted and transfected into the nucleus of the cells. Cell culturing of these transfected cells lead to secretion of EVs containing gene of interest

Genetic engineering of EVs is an easy and convenient approach for modification to deliver new characteristics. This approach was used in five studies [56–61]. Alvarez-Erviti and coworkers used genetic engineering to deliver siRNA into the mouse brain through systemic injection of targeted EVs [59]. Figure 10 shows the genetic engineering approach for the synthesis of engineered EVs. Using this approach parent’s cells produce a homogenous population of EVs loaded with the fabricated or desired property. No cell toxicity was reported using this modification method. The versatility of this method is that it allows the loading of RNA, DNA, and peptides of choice into the EVs. Choice of specific EVs derived cells is an associated limitation of this method. Tumour derived EVs might interplay in the premetastatic niche and initiate negative effects. Although studies are not enough to decide the negative effects of these EVs derived from tumour cells. Another limitation is the use of the adenoviral gene; this is because in some cases humans have shown immune response.

**Fig. 11:**
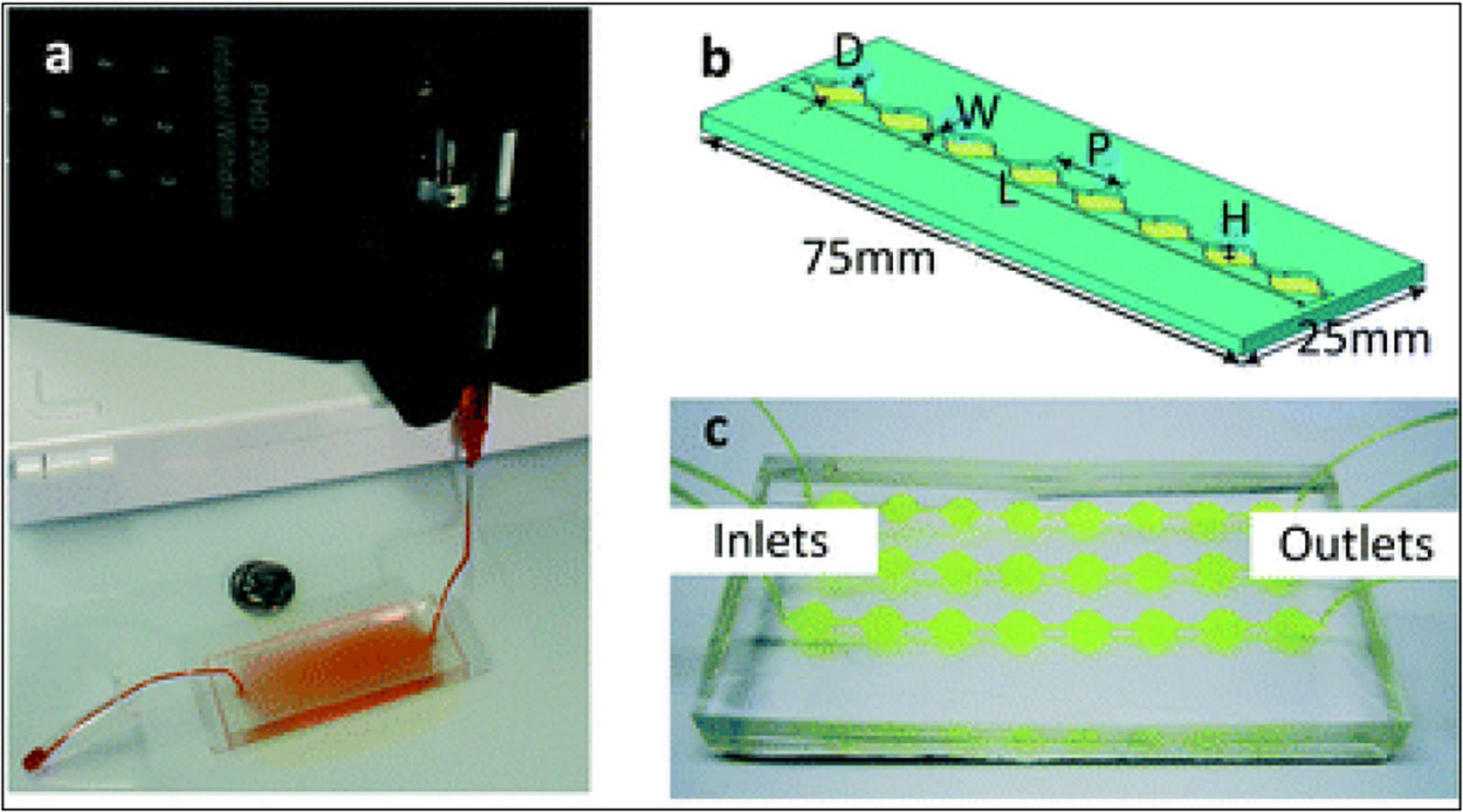
**a** Microfluidics approach of microfluidic EVs isolation featured. The pump-assisted entry of EVs into micro channels of the device enable capturing of EVs that might be helpful in loading of the desired drug/cargo. **b** Single channel Exochip (**c**) Experimental setup using a 3 channels Exochip. (Adapted from Reference [64] under open access article distributed under the Creative Commons Attribution 3.0 Unported Licence)

Two authors have used a recently developed technique named microfluidics for EVs modification [60, 61]. The design of the microfluidics platform is shown in Fig. 11. In one study, microfluidics was utilized in the synthesis of synthetic EVs containing mRNAs, proteins with a shape similar to those used in drug delivery [60]. In another study, authors used a microfluidics approach by integrating cell harvesting, antigenic modifications, and photo-release of surface-engineered EVs on one polydimethylsiloxane (PDMS) platform [61]. Microfluidics technology is a recent approach for the modification of EVs with desired cargo. Due to its low cost, time saving, short processing time, preservation of EVs integrity, stability maintenance, high throughput precision, and low sample volume, this loading approach is gaining attention nowadays in EVs therapeutics. Another advantage of microfluidics is the opportunity to couple with automation that is helpful in mass-scale cargo loading for clinical settings. This technique offers a clear benefit over other cargo loading methods. The only disadvantage with this method is that sometimes polymeric material or non-EVs ingredient causes co-precipitation along with EVs.

## Discussion

Through this detailed systematic analysis of the pioneer publications we gathered a global picture of the EVs modification approaches that can be exploited for efficient drug delivery. The significant increase in number of publications related to EVs modification; prove their efficiency and capacity to carry out a vast array of therapeutic molecules in treatment of various diseases. These tailoring approaches further exploited the reprogramming of EVs, loading of desired targeted drugs, and tracking of EVs for diagnostics. Table 1 demonstrates the application of cargo-loaded EVs in the treatment of various human diseases. Considering the findings of the present study, the freeze–thaw method is the most recommended approach for the modification of EVs, followed by co-incubation.

The present systematic review analysis showed that freeze-thawing is a simple, easy, and time efficient physicochemical mechanism that can produce hybrid EVs due to membrane fusion. This process relies on the disruption of the lipid bilayer membrane due to the formation of temporary ice crystals [46]. In other words, this process could be described as the removal of water molecules present within hydrophilic moieties of the lipid bilayer membrane as the EVs freeze, thereby disrupting the structure of the EVs and initiating phase changes and reconstitution of the membrane. The authors of the cited studies successfully reprogrammed EVs by utilizing the freeze–thaw process [46–53].

This process involves several cycles of freezing (in liquid nitrogen, − 210 °C) and thawing (at room temperature), which disturbs the EVs membrane integrity, thereby causing the cargo (liposomes, drugs, nano-particles, and peptides) to enter inside or fused with membrane as shown by recently published study (Fig. 9) [46]. In Table 2 various modification approaches were described that acted as a revolutionized tool in biotechnology. EVs were also been exploited as intervention therapy with the advancement in biotechnology, several clinical trials are various phases that decipher the potential of these EVs in treatment of several diseases as shown in Table 3.

**Table 2:** Modifications of extracellular vesicles and its role in nano-biotechnology

EVs Type	Nanotechnological Modification	Application	Loading Method
Exosomes from mesenchymal stem cells	Glucose-coated gold nanoparticles (NPs)	In vivo neuroimaging	Co-incubation
Exosome from lung cancer or fibroblasts	Gold NPs and doxorubicin	Lung cancer treatment	Co-incubation
EVs from breast adenocarcinoma	MOF NPs. NPs matrix contained gelonin	Inhibit adenocarcinoma growth	Sonication and extrusion
Exosomes from Hela cells	MOF NPs	Hela cells	Co-incubation
EVs from KB cells	ZnO NPs	Cytotoxic effect against KB cells	Co-incubation
EVs from endothelial, cancer and stem cell lines	Porphyrins	To improve photodynamic therapy	Electroporation, extrusion, saponin-assisted and dialysis
Exosomes from embryonic stem cells	Paclitaxel	Glioma therapy	Co-incubation
Milk-derived exosomes		To reduce paclitaxel’s side effects	Co-incubation
Exosomes from macrophages		To overcome MDR in cancer cells	Co-incubation, electroporation and sonication
Exosomes from brain cell lines		To treat brain tumor	Co-incubation
EVs from prostatic cancer		Cytotoxic effect against prostate cancer	Co-incubation
Exosomes from human colorectal carcinoma	Doxorubicin	Antiproliferative effect in colorectal cancer	Dialysis
Exosomes from breast cancer		To treat breast and ovarian cancer	Electroporation
Exosomes from breast cancer		To reduce cardiotoxicity of doxorubicin	Electroporation
Exosomes from 4T1, MCF-7, and PC3 cell line		Breast cancer	Co-incubation
Exosomes from mouse immature dendritic cells		For targeted delivery of chemotherapeutic	Electroporation
Milk-derived exosomes	Curcumin	Cervical cancer	Co-incubation
Exosomes from lymphoma cells		Activate myeloid cells in vivo	Co-incubation
Plant exosomes		Colon cancer	
Milk-derived exosomes	Paclitaxel, Docetaxel, Withaferin A and curcumin	Targeting and therapy of lung cancer cells	Co-incubation
Milk-derived exosomes	Celastrol	Inhibition of Hsp90 and NF- κB activation pathways in lung cancer	Co-incubation
EVs from lung cancer	Oncolytic adenovirus and paclitaxel	Enhance immunogenicity in lung cancer	Co-incubation
Exosomes from HEK 293 cells	siRNA	Efficient delivery of siRNA in cancer cells	Electroporation
Exosomes from HEK 293 cells	Polo-like kinase 1 (PLK-1) siRNA	Silencing PLK-1 gene in bladder cancer cells	Electroporation
Exosomes from HEK 293 and MCF-7 cells	siRNA, miRNA and ssDNA ^b^	Oncogene knockdown	Sonication
Plasma-derived EVs	miRNA cel-39	Promote apoptosis of hepatocellular carcinoma	Electroporation

(Adapted from Ref No. [65]) under the Creative Commons Attribution License

**Table 3:** EVs based intervention clinical trials (Source: clinicaltrials.gov)

EVs Source	Condition	Drug	Administration Route	Dose Reported	Phase	Study Identifier
MSCs	Cerebrovascular disorders/stroke	miR-124	i.v	200 µg protein	1/2	NCT03384433
MSCs	Alzheimer Disease	No	Nasal drip	5 μg–20 μg	1/2	NCT04388982
MSCs	Periodontitis	No	Local	Not reported	early 1	NCT04270006
MSCs	Neuralgia	No	i.v. epineurally	45 mg, 15 mg	n/a	NCT04202783
MSCs	Depression	No	i.v	21 million cells	n/a	NCT04202770
MSCs	Diabetes Mellitus Type 1	No	i.v	1.2 × 1010–1.51 × 1010 particles/kg	2/3	NCT02138331
MSCs	Chronic Ulcer	No	Topical	Not reported	1	NCT04134676
Plasma	Ulcer	No	Topical	Not reported	Early 1	NCT02565264
MSC	Dystrophic Epidermolysis Bullosa	No	Topical	Not reported	1/2	NCT04173650
MSCs	Multiple Organ Failure	No	i.v	150 mg once a day for 14 times	n/a	NCT04356300
MSCs	Healthy	No	Inhalation	2 × 108–20 × 108 particles/3 ml	1	NCT04313647
Plant	Colon Cancer	Curcumin	Oral	Not reported	1	NCT01294072
Plant	Polycystic Ovary Syndrome	No	Oral	Not reported	n/a	NCT03493984
Grape	Head and Neck Cancer Oral Mucositis	No	Oral	Not reported	1	NCT01668849
DCs	Non Small Cell Lung Cancer	MHC class I- class II- cancer antigens	i.v	53–2422 μg protein/injection	2	NCT01159288
MSCs	Pancreatic Adenocarcinoma	KRAS G12D siRNA	i.v	Days 1, 4, and 10 (dose not reported)	1	NCT03608631
MSCs	SARS-CoV-2 pneumonia	No	Inhalation	2 × 108 particles/3 mL	1	NCT04276987
MSCs	SARS-CoV-2 pneumonia	No	Inhalation	0.5 × 1010–2 × 1010 particles/3 ml	1/2	NCT04491240
Bone marrow	SARS-CoV-2 pneumonia	No	i.v	Not reported	2	NCT04493242
Human amniotic fluid	SARS-CoV-2 pneumonia	No	i.v	2 × 1010–5 × 1010 particles	1/2	NCT04384445
MSCs	Dry Eye	No	Eye drop	10 µg/drop	1/2	NCT04213248
MSCs	Macular Holes	No	Drop	50 μg or 20 μg	Early 1	NCT03437759

(Adapted from Ref No. [66]) under the Creative Commons Attribution License 4.0

The present study has some limitations that warrant consideration. Modifications of EVs through various other approaches have not been addressed in this systematic review. Additional clinical trials are needed to further validate the authenticity and global acceptability of specific modification approaches. However, the present study summarizes the most commonly used tailoring approaches for EVs modifications with future perspectives in therapeutics and diagnostics.

## Conclusion

Extracellular vesicles exhibit promising therapeutic and diagnostic uses in biomaterials. These tailored nanovesicles can be loaded with desired biomolecules, such as proteins, lipids, nucleic acids, and drugs, using different modification approaches to obtain functionalized EVs. These functionalized EVs can be exploited in the treatment of various diseases, like cancer, inflammation-associated morbidities, and other cellular regeneration processes. Moreover, these loaded and functionalized EVs can also be investigated to elucidate the cellular uptake mechanism and for screening as a diagnostic tool. Several modification methods are available at present; however, the freeze–thaw and co-incubation approaches are preferred. Further clinical studies are needed to support specific modification approaches.

## Data Availability

Not applicable.
